# Supplementary material for: Protein structure and folding pathway prediction based on remote homologs recognition using PAthreader
Source: Commun Biol. 2023 Mar 4;6:243. doi: 10.1038/s42003-023-04605-8 (PMC9985440; doi:10.1038/s42003-023-04605-8)
Supplement: Supplementary file 2 — Supplementary Information [file 42003_2023_4605_MOESM2_ESM.pdf]

Supplementary Information

Protein structure and folding pathway prediction based on remote homologs recognition using PAthreader

Kailong Zhao[1], Yuhao Xia[1], Fujin Zhang[1], Xiaogen Zhou[1], Stan Z. Li[2*], and Guijun Zhang[1*]

[1] College of Information Engineering, Zhejiang University of Technology, HangZhou 310023, China; [2] AI Lab, Research Center for Industries of the Future, Westlake University, Hangzhou 310024, Zhejiang, China.

*Correspondence should be addressed to Guijun Zhang (zgj@zjut.edu.cn) and Stan Z. Li (Stan.ZQ.Li@westlake.edu.cn).

Supplementary Note

Note S1. Structure clustering in master structure database construction.

We used a greedy incremental clustering method similar to CD-HIT[1] to construct the master structure database. The clustering algorithm sorts the structures according to the sequence length from long to short, and processes them in the order from longest to shortest. The first structure is used as the first cluster representative structure. Each structure of the remaining structures is then compared to a previously found representative structures, and is classified as redundant or representative based on whether it is similar to one of the existing representative structures. After the structure is grouped into representative, it does not need to be compared with other representatives. The similarity of the two structures is measured by the TM-score calculated by TM-align. When the reference structures are different, two scores are calculated for the similarity of the two structures. Both scores are above 0.8 to be classified as one group. Therefore, the lengths of structures in the same cluster are all in the range [0.8*L, 1.25*L], where L is the length of the representative structure.

Supplementary Figures

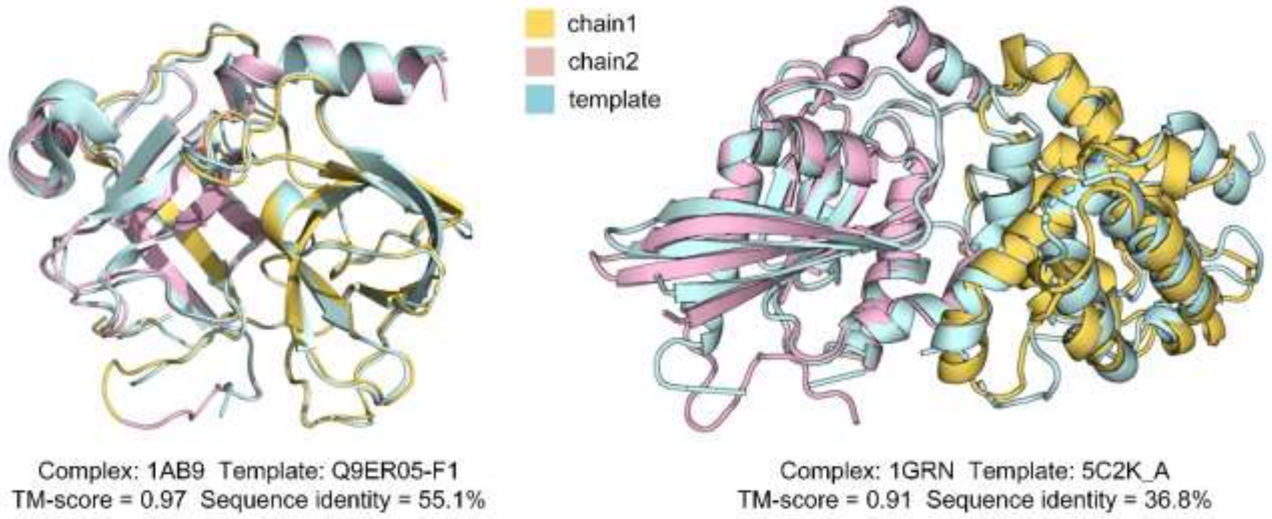

Figure S1. Monomeric templates (blue) detected by PAthreader for complexes 1AB9 and 1GRN.

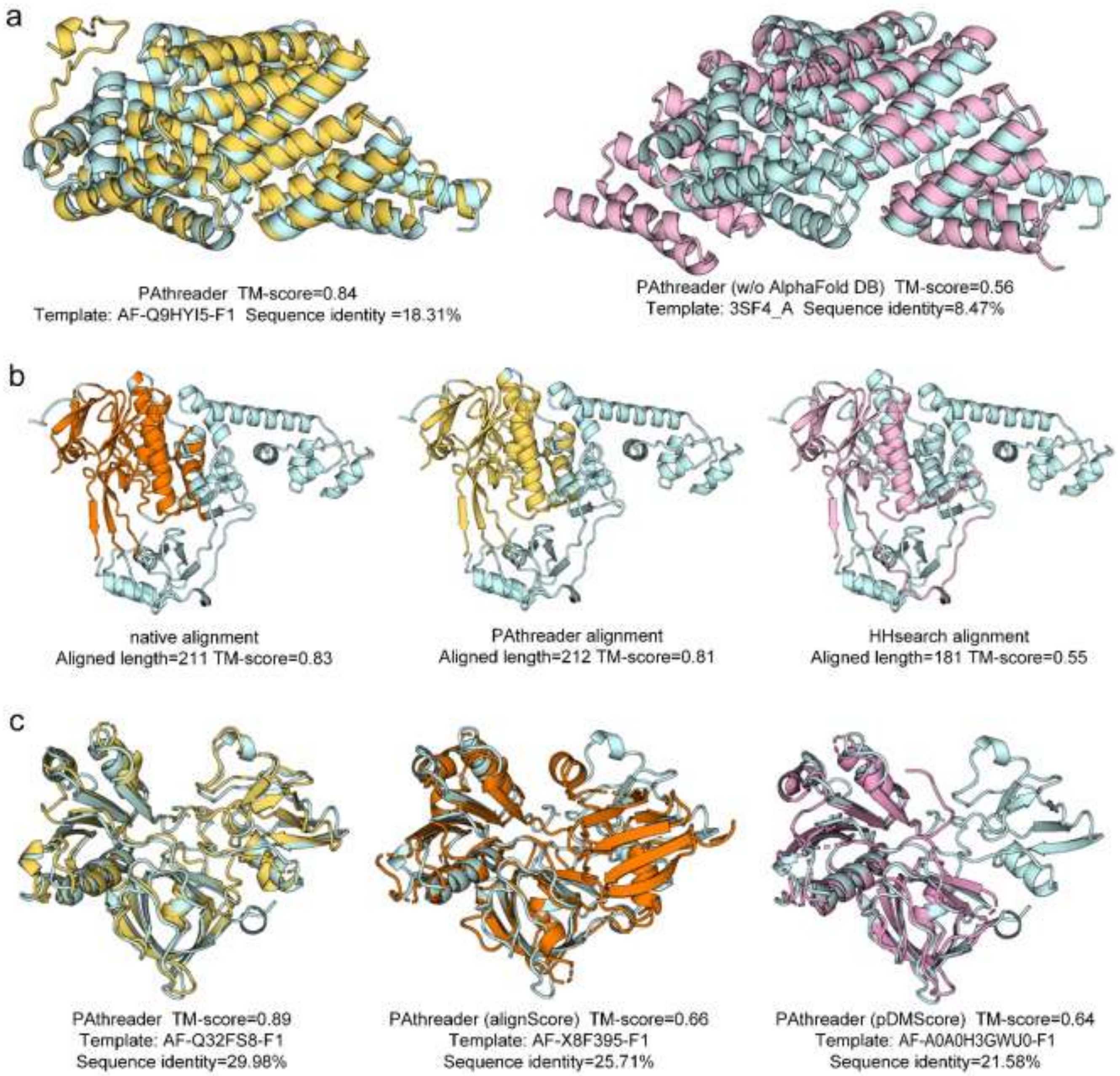

Figure S2. a An illustrative example from 1HZ4_A, showing the structure superposition of the PAthreader template (yellow) and PAthreader (w/o AlphaFold DB) template (pink) with the native structure (blue). **b** An illustrative example from 2CUL_A shows that PAthreader and HHsearch provide different alignment results for the same structure. 3CES_A (blue) is identified as the best template structure for the query target using both PAthreader and HHsearch. The native alignment (orange) represents the result obtained by comparing the identified structure with the native structure through TM-align. The PAthreader alignment (yellow) is obtained by threading the sequences into the identified structures based on the sequence alignment provided by the three-track alignment, and the HHsearch alignment (pink) is generated by Hidden Markov-constructed profiles comparison. **c** An illustrative example from 2PIA_A, showing the structure superposition of the PAthreader template (yellow), PAthreader (alignScore) template (orange) and PAthreader (pDMscore) template (pink) with native structure (blue).

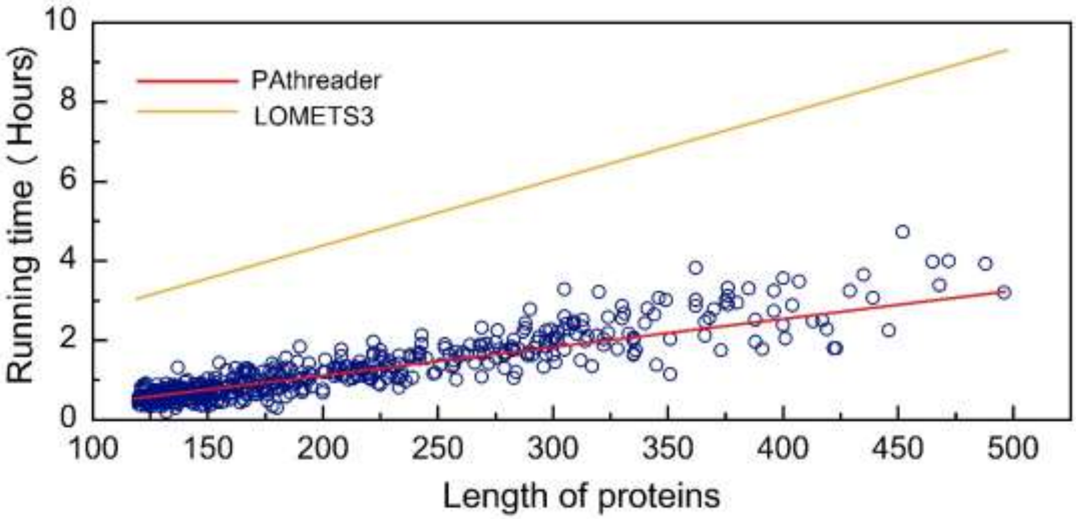

Figure S3. The running time of PAthreader and LOMETS3 to search templates. The x-axis is the length of protein and the y-axis is the running time in hours. The running time of LOMETS3 are taken from the supporting information of LOMETS3[3], and that of PAthreader is linearly fitted according to the actual running time on 551 test proteins.

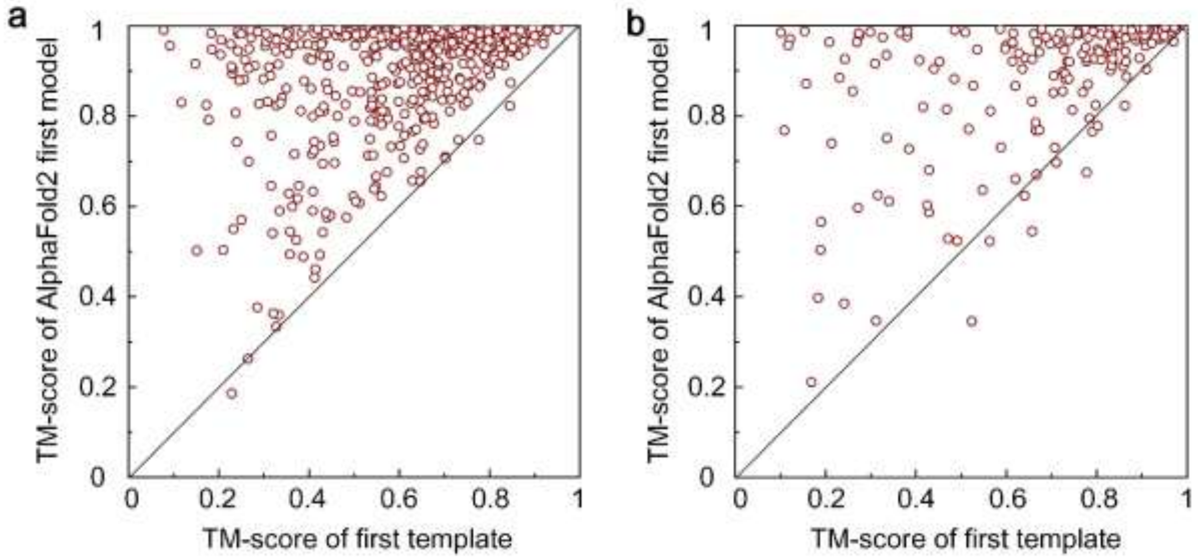

Figure S4. a, b The relationship between model accuracy and template quality of AlphaFold2 on 551 test proteins and 186 cameo proteins, respectively. In the modeling of AlphaFold2, templates with ≥ 30% sequence identity were removed. We selected the first template of HHsearch to compare with AlphaFold2's first model. The first template is obtained by ranking Sum_probs values of HHsearch templates. Sum_probs is the sum over the posterior probabilities of all aligned pairs of match states, which usually correspond to the template with the highest accuracy[2].

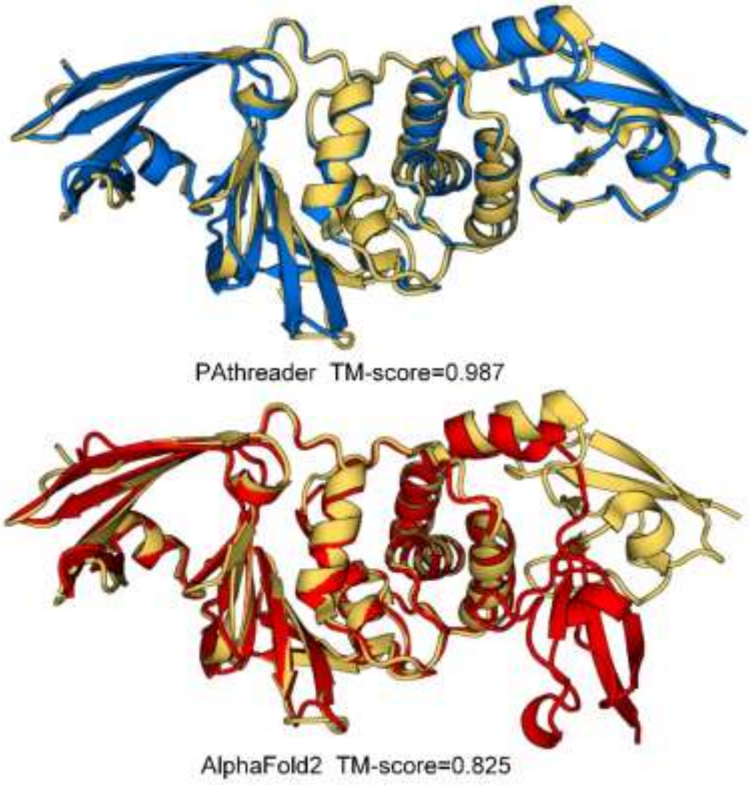

Figure S5. An illustrative example from Papain-like proteinase of SARS-CoV-2 virus, showing the structure superposition of the PAthreader model (blue) and AlphaFold2 model (red) with the native structure (yellow).

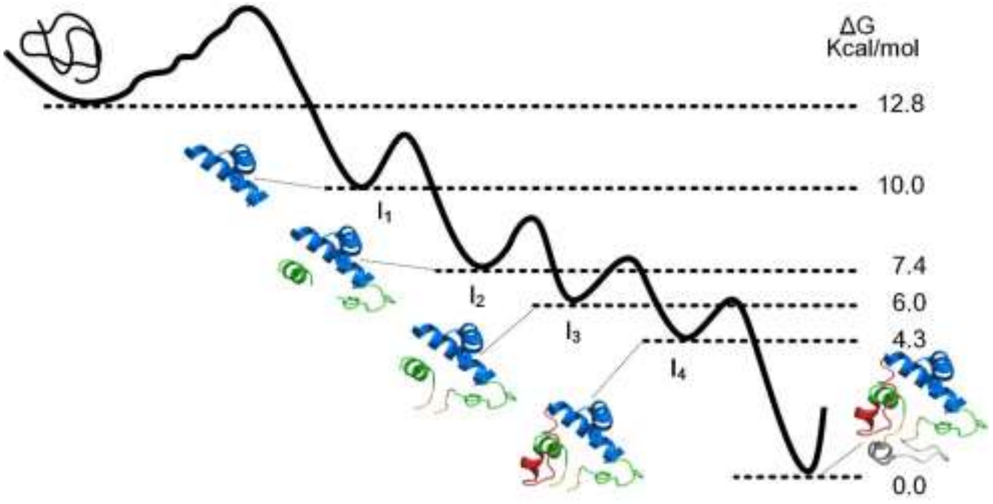

Figure S6. The second experimental pathway of horse heart cytochrome c is determined by hydrogen exchange (HX) pulse labeling and nuclear magnetic resonance (NMR). The literature show that cytochrome c unfolds by stepping uphill through a ladder form. First, the grey bottom loop of cytochrome c unfolded alone, then grey + red unfolded, then these two + yellow unfolded, then these three + green, and finally N- and C-terminal blue helical segments unfolded[4,5]. It contains 4 folding intermediates, I_1, I_2, I_3 and I_4.

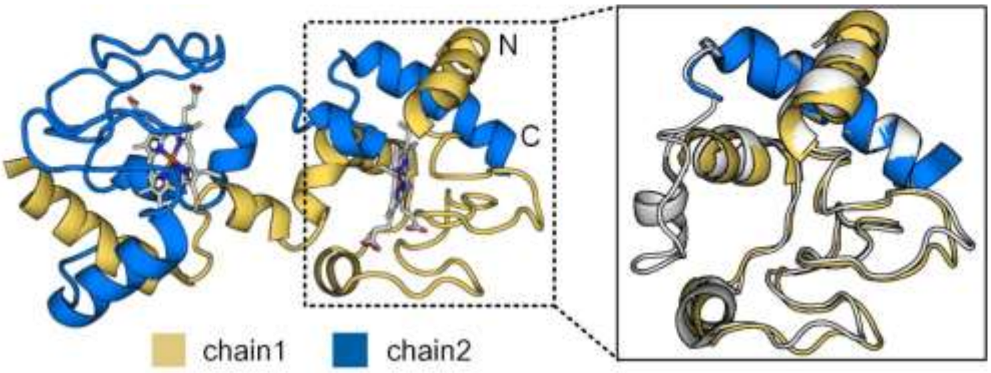

Figure S7. Crystal structure of a domain-swapped dimer of yeast iso-1-cytochrome c with omega-undecylenyl-beta-D-maltopyranoside (PDB ID: 5KLU). The solid line box is the partial superposition of 5KLU and structure of horse heart cytochrome c (grey).

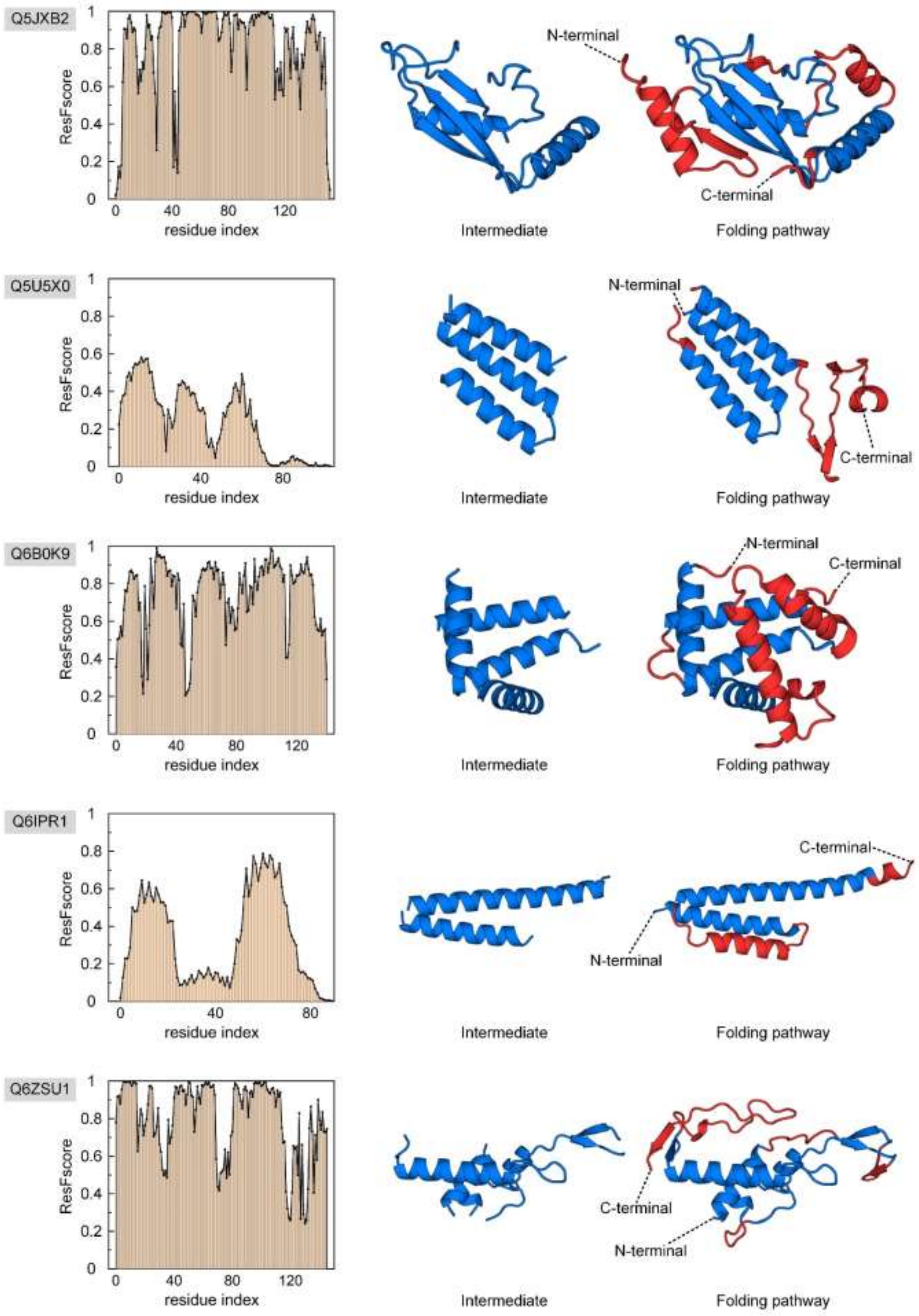

Q5JXB2
ResFscore
residue index
N-terminal
C-terminal
Intermediate
Folding pathway
Q5U5X0
ResFscore
residue index
N-terminal
C-terminal
Intermediate
Folding pathway
Q6B0K9
ResFscore
residue index
N-terminal
C-terminal
Intermediate
Folding pathway
Q6IPR1
ResFscore
residue index
C-terminal
N-terminal
Intermediate
Folding pathway
Q6ZSU1
ResFscore
residue index
C-terminal
N-terminal
Intermediate
Folding pathway

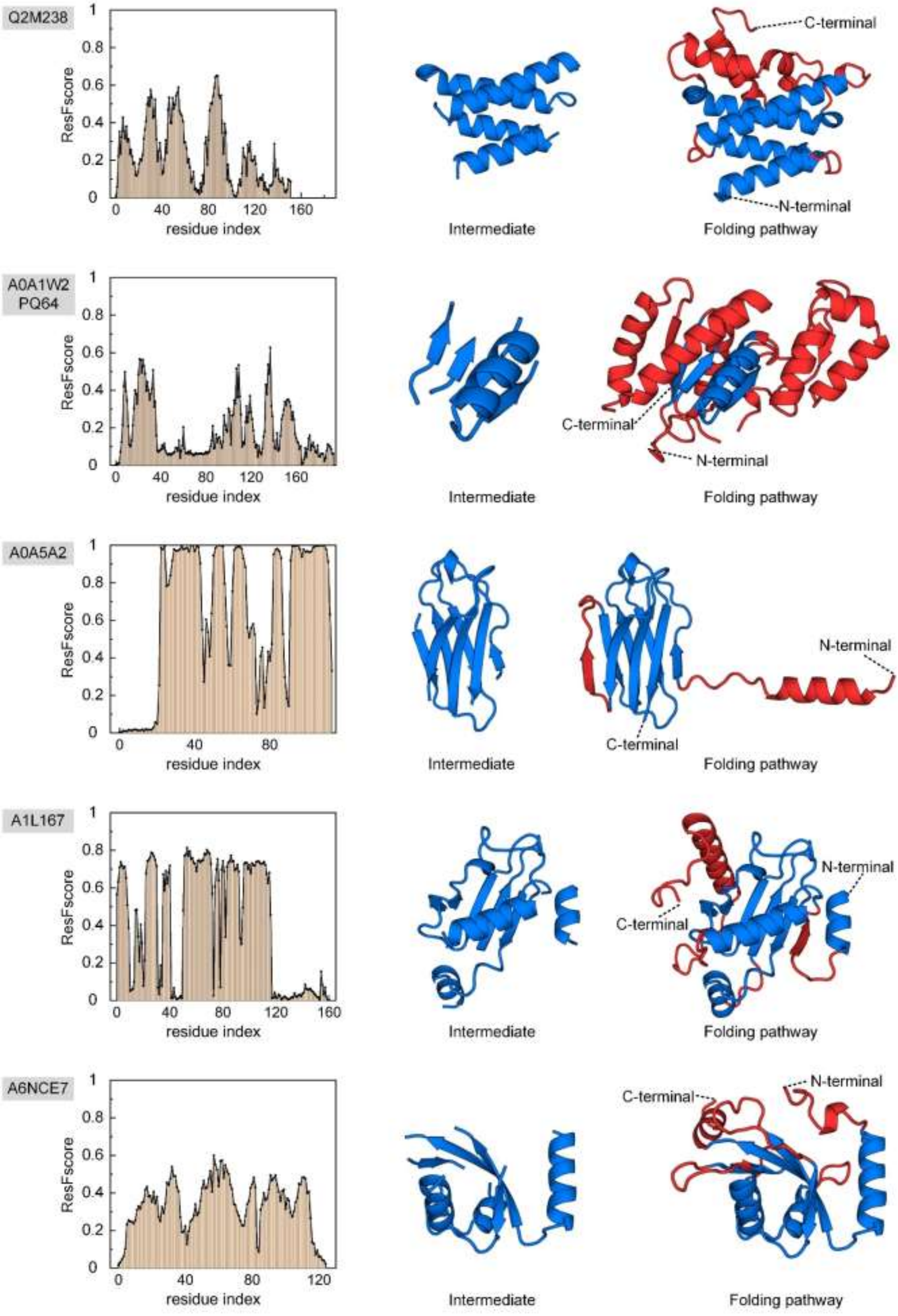

Q2M238
ResFscore
residue index
Intermediate
Folding pathway
C-terminal
N-terminal
A0A1W2 PQ64
ResFscore
residue index
Intermediate
Folding pathway
C-terminal
N-terminal
A0A5A2
ResFscore
residue index
Intermediate
Folding pathway
N-terminal
C-terminal
A1L167
ResFscore
residue index
Intermediate
Folding pathway
N-terminal
C-terminal
A6NCE7
ResFscore
residue index
Intermediate
Folding pathway
C-terminal
N-terminal

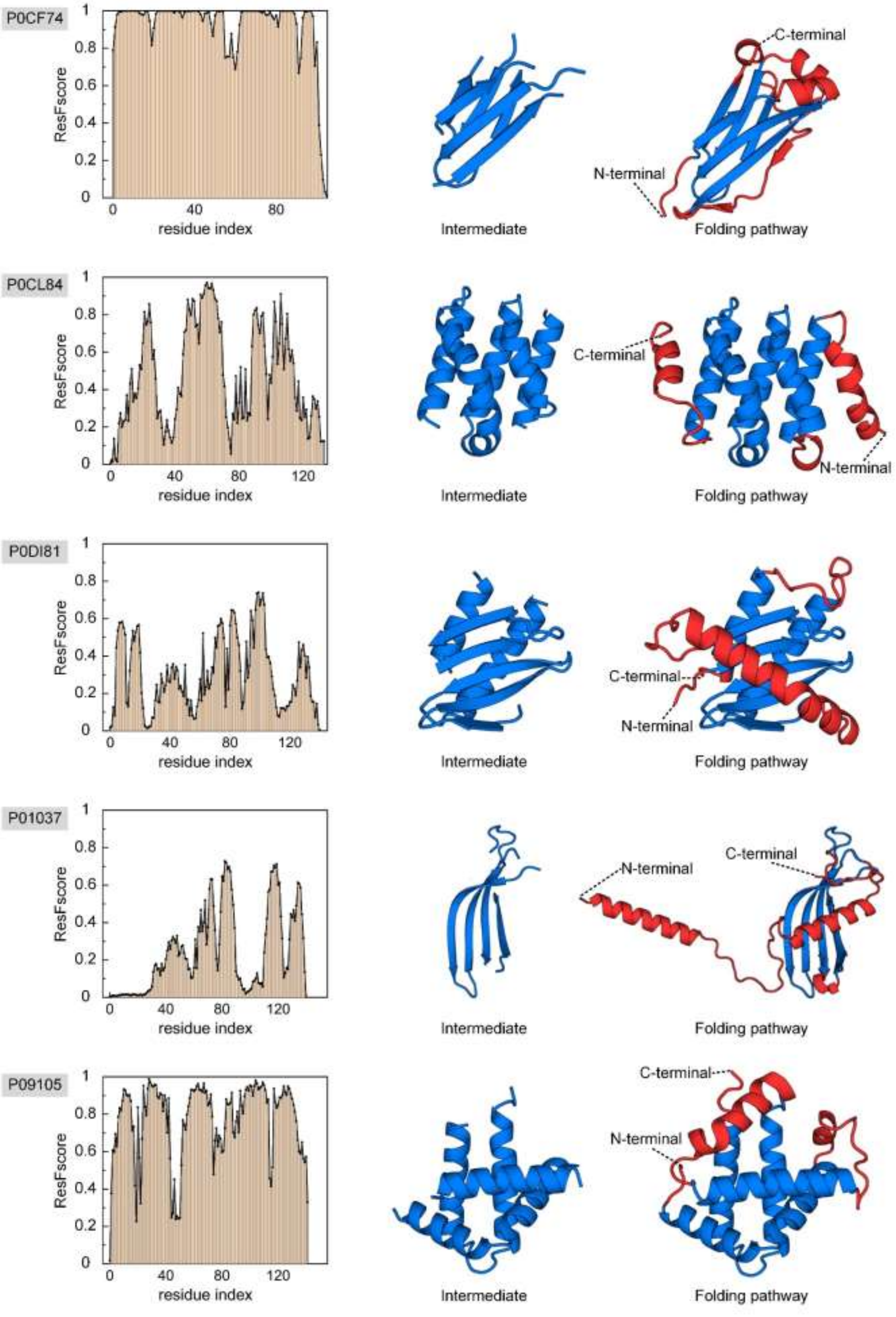
P0CF74
ResFscore
residue index
Intermediate
Folding pathway
C-terminal
N-terminal
P0CL84
ResFscore
residue index
Intermediate
Folding pathway
C-terminal
N-terminal
P0DI81
ResFscore
residue index
Intermediate
Folding pathway
C-terminal
N-terminal
P01037
ResFscore
residue index
Intermediate
Folding pathway
N-terminal
C-terminal
P09105
ResFscore
residue index
Intermediate
Folding pathway
C-terminal
N-terminal

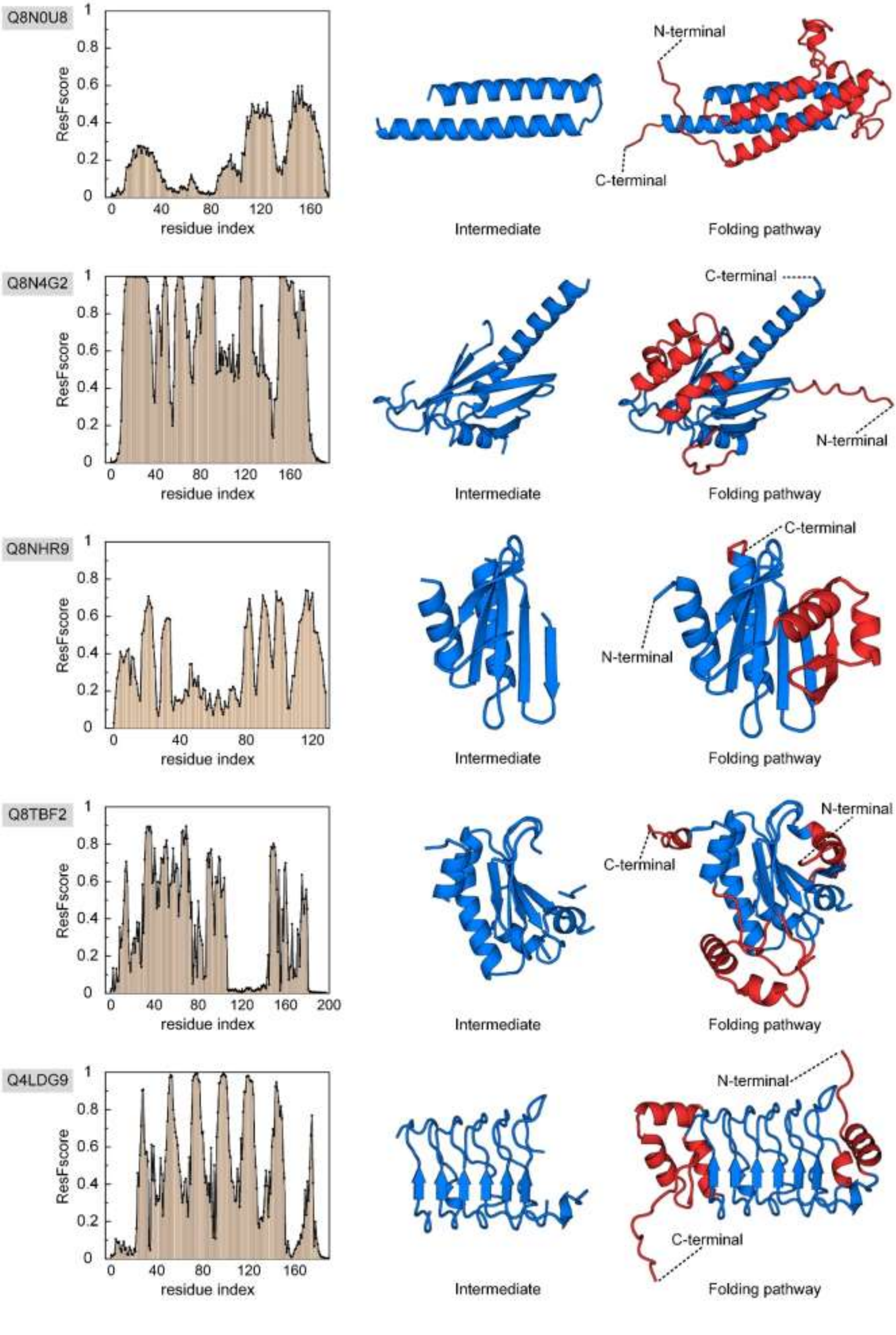
Q8N0U8
ResFscore
residue index
Intermediate
Folding pathway
N-terminal
C-terminal
Q8N4G2
ResFscore
residue index
Intermediate
Folding pathway
C-terminal
N-terminal
Q8NHR9
ResFscore
residue index
Intermediate
Folding pathway
C-terminal
N-terminal
Q8TBF2
ResFscore
residue index
Intermediate
Folding pathway
N-terminal
C-terminal
Q4LDG9
ResFscore
residue index
Intermediate
Folding pathway
N-terminal
C-terminal

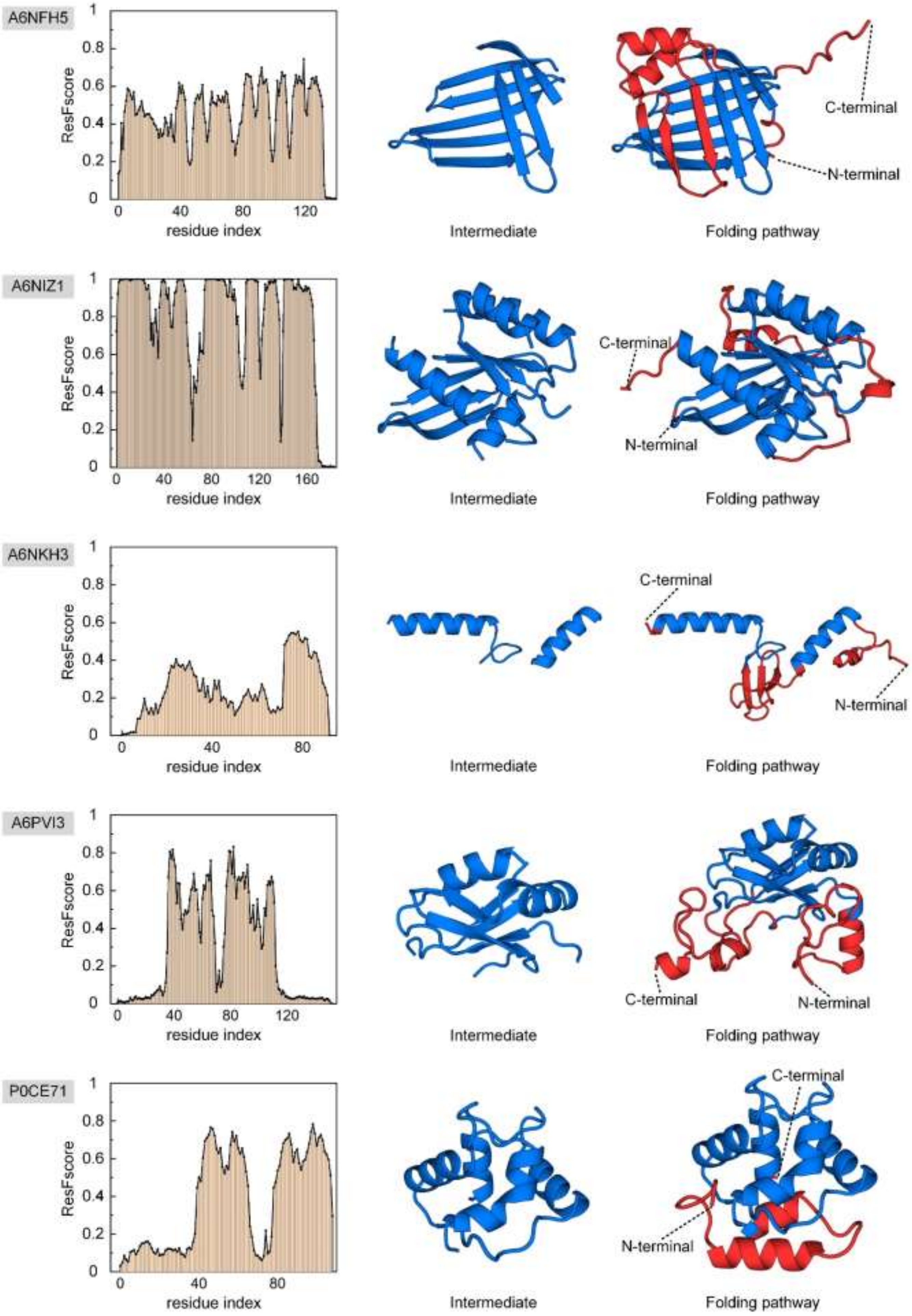

A6NFH5
ResFscore
residue index
Intermediate
Folding pathway
C-terminal
N-terminal
A6NIZ1
ResFscore
residue index
Intermediate
Folding pathway
C-terminal
N-terminal
A6NKH3
ResFscore
residue index
Intermediate
Folding pathway
C-terminal
N-terminal
A6PVI3
ResFscore
residue index
Intermediate
Folding pathway
C-terminal
N-terminal
P0CE71
ResFscore
residue index
Intermediate
Folding pathway
C-terminal
N-terminal

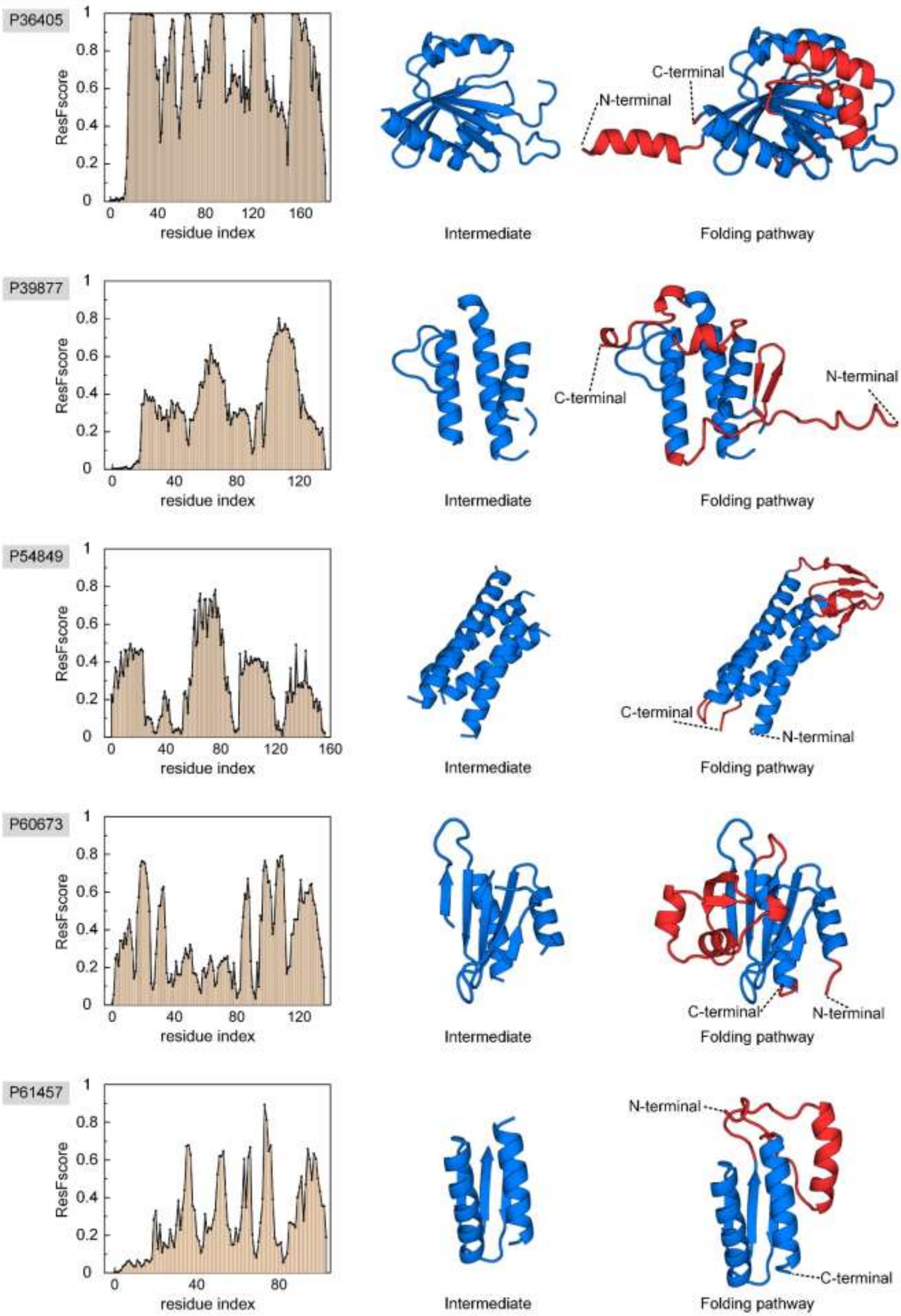
P36405
ResFscore
residue index
Intermediate
Folding pathway
C-terminal
N-terminal
P39877
ResFscore
residue index
Intermediate
Folding pathway
C-terminal
N-terminal
P54849
ResFscore
residue index
Intermediate
Folding pathway
C-terminal
N-terminal
P60673
ResFscore
residue index
Intermediate
Folding pathway
C-terminal
N-terminal
P61457
ResFscore
residue index
Intermediate
Folding pathway
N-terminal
C-terminal

Figure S8. The folding pathways of 30 human proteins were determined by PAthreader. The residue frequency distributions are shown on the left, the folding intermediate are shown in the middle, and the folding order are shown on the right. The folding order is blue and then red.

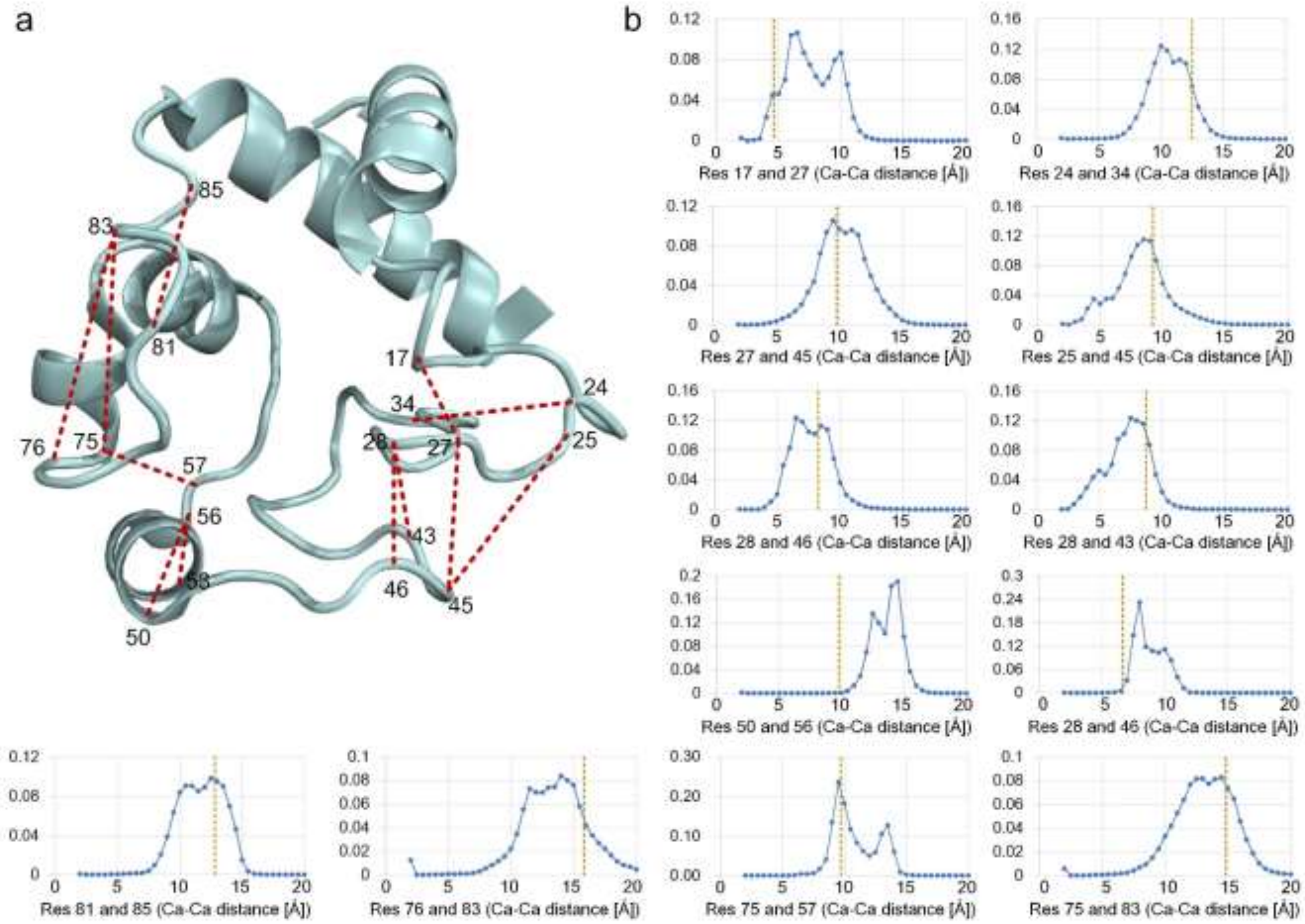

Figure S9. a 3D structure of oxidized horse heart cytochrome c. **b** Multi-peak distance distribution of flexible regions protein predicted by DeepMDisPre. The yellow dotted line is the true distance corresponding to the 3D structure. The figure presents 12 predicted cytochrome c residue pairs with multiple local maxima that were significantly visible, which distributed in the flexible region except three helices.

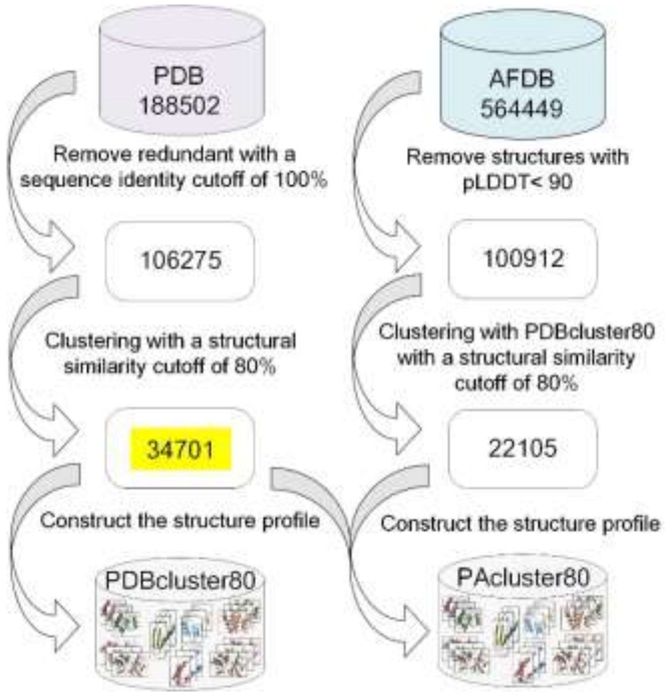

Figure S10. Schematic of the construction of the master structure database. PAcluster80 is constructed with 56,805 clusters consisting of 106,275 PDB structures and 100,912 AlphaFold DB structures. The structural profiles are extracted from the structural classes.

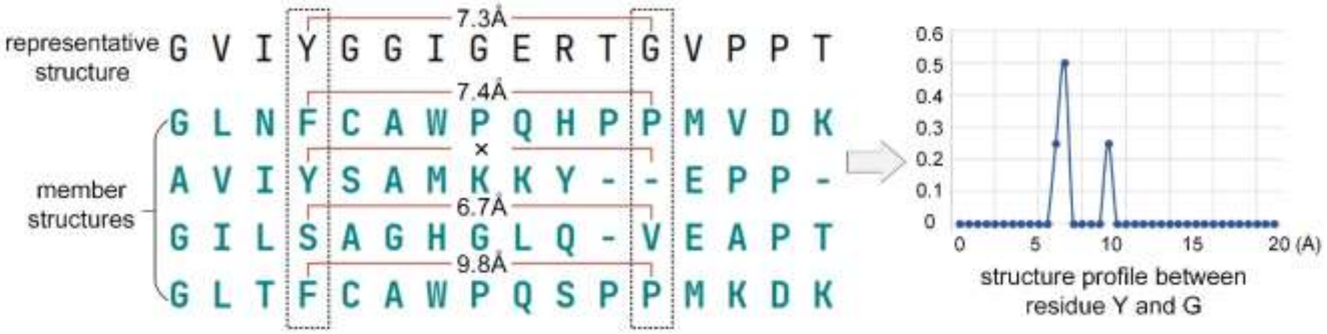

Figure S11. Schematic of the construction of structure profiles from structure classes in the master structure database PAcluster80. The member structures of the clusters are globally aligned with the center structure by TM-align. The distance range (2-20 Å) was then divided into 36 bins with a size of 0.5 Å, plus one bin for residue pair distances ≥ 20 Å. The number of times of falling into the bin divided by the total was taken as the probability. The residues pairs with gaps are not included in the total.

Supplementary Tables

Table S1. Summary of the results of the ablation experiments. The results are obtained by computing the TM-score of the first template.

	(0.9, 1.0]	(0.7, 0.9]	(0.5, 0.7]	(0.0, 0.5]	All
Contribution of AlphaFold DB					
PAthreader	**0.899**	**0.787**	**0.568**	**0.424**	**0.725**
PAthreader (w/o AlphaFold DB)	0.898	0.775	0.521	0.324	0.702
HHsearch	0.840	0.718	0.476	0.272	0.646
LOMETS3	0.868	0.754	0.534	0.342	0.689
Contribution of pDMScore					
PAthreader	**0.899**	**0.787**	**0.568**	**0.424**	**0.725**
PAthreader (alignScore)	0.896	0.781	0.562	0.408	0.718
PAthreader (pDMScore)	0.878	0.774	0.557	0.422	0.712

Table S2. TM-score of template recognition on 551 tested proteins. Tested proteins were divided into four subsets (0-0.5, 0.5-0.7, 0.7-0.9 and 0.9-1) based on TM-score of the best template of targets in PDB. Bold text highlights the best result in each category.

	(0.9, 1.0]	(0.7, 0.9]	(0.5, 0.7]	(0.0, 0.5]	All
	num (58)	num (321)	num (149)	num (23)	num (551)
PAthreader	**0.899**	**0.787**	**0.568**	**0.424**	**0.725**
HHsearch	0.840	0.718	0.476	0.272	0.646
LOMETS3	0.868	0.754	0.534	0.342	0.689
SPARKS-X	0.851	0.736	0.505	0.326	0.669
MUSTER	0.854	0.737	0.500	0.327	0.668
CEthreader	0.859	0.740	0.507	0.322	0.672
EigenTHREADER	0.841	0.727	0.488	0.324	0.656

Table S3. Summary of the results of top 10 public servers on the three-month CAMEO blind test (https://www.cameo3d.org/modeling/3-months/difficulty/all/?to_date=2022-07-09).

Server name	Targets		Average LDDT	
	All	#Modeled	All	#Modeled
PAthreader	189	189	84.4	84.4
SADA	189	189	83.9	83.9
ZJUT-DeepAssembly	189	183	81.4	84.0
MultiDFold	189	189	81.2	81.2
AIRFold	189	181	80.2	83.8
MEGA-EvoGen	189	175	78.0	84.3
ManiFold	189	172	74.9	82.3
IntFOLD7	189	169	72.3	80.8
RoseTTAFold	189	175	68.2	73.6
pureAF2_notemp	189	142	63.2	84.2

3-months - [2022-04-15 - 2022-07-09] - "All targets" dataset

Server Name (Common Subset - Start Comparison)	Avg. response time (hh:mm:ss)	Targets				Average lDDT	
		#Submitted	#Modeled	#Submitted Oligo	#Modeled Oligo	All	Modeled
PAthreader	56:47:48	189	189	58	0	84.4	84.4
SADA	65:06:46	189	189	58	0	83.9	83.9
ZJUT-DeepAssembly	69:44:45	189	183	58	0	81.4	84.0
MultiDFold	76:56:34	189	189	58	0	81.2	81.2
AIRFold	20:22:30	189	181	58	0	80.2	83.8
MEGA-EvoGen	64:33:26	189	175	58	0	78.0	84.3
ManiFold	69:23:44	189	172	58	0	74.9	82.3
IntFOLD7	31:36:32	189	169	58	0	72.3	80.8
RoseTTAFold	19:17:58	189	175	58	0	68.2	73.6
BestSingleStructuralTemplate	04:11:21	189	171	58	0	64.4	71.2
pureAF2_notemp	24:12:41	189	142	58	0	63.2	84.2

Table S4. Summary of the results of 17 proteins of SARS-CoV-2 virus.

	Structure modelling		Template recognition	
	PAthreader	AlphaFold2	PAthreader	HHsearch
Helicase	0.968	0.969	0.945	0.945
Proteinase 3CL-PRO	0.981	0.964	0.993	0.985
nucleocapsid protein	0.962	0.917	0.961	0.749
Non-structural protein 7	0.953	0.861	0.952	0.573
Uridylate-specific endoribonuclease	0.989	0.967	0.975	0.081
ORF7a	0.943	0.943	0.924	0.924
Non-structural protein 9	0.877	0.873	0.841	0.789
Papain-like proteinase	0.995	0.991	0.995	0.954
Non-structural protein 10	0.171	0.176	0.191	0.170
Papain-like proteinase	0.987	0.825	0.985	0.861
ORF3a	0.812	0.800	0.355	0.186
nucleocapsid protein	0.974	0.955	0.836	0.836
Non-structural protein 8	0.580	0.390	0.419	0.575
ORF8	0.410	0.749	0.270	0.208
Envelope Protein Transmembrane Domain	0.607	0.602	0.599	0.610
NSP1	0.947	0.947	0.356	0.896
Papain-like proteinase	0.874	0.870	0.731	0.732
Average TM-score	0.825	0.812	0.725	0.651

Table S5. Summary of the results of protein folding pathway exploration. 30 human proteins whose native structures have not been determined by biological experiments were labeled with their UniProt accession. The first template is labelled by PDB ID or AlphaFold DB ID. The last column represents the proportion of templates used to identify intermediates in the different pfam families (only the top three families with the highest proportions are listed), where templates from the PDB were used for the statistics (most of the structures from the AlphaFold DB were not assigned to pfam family).

	Protein	First template	Residue range of folding intermediate	pfam family
7 proteins (crystal structures and folding pathways determined by biological experiments)				
1	1I5T	AF-P99999-F1	3-13, 61-67, 88-101	Cytochrom_C (49.7%)<br>S1-P1_nuclease (5.7%)<br>H_PPase (4.5%)
2	1BE9	5HF4_A	12-29, 36-92	PDZ (69.2%)<br>FAD_binding_3 (4.6%)<br>Amino_oxidase (4.6%)
3	2LZM	3FI5_A	1-13, 65-164	Phage_lysozyme (80.8%)<br>7tm_1 (11.1%)<br>Pesticin (2.0%)
4	1DKT	AF-B0G102-F1	3-25, 51-68	Pkinase (63.5%)<br>PK_Tyr_Ser-Thr (18.6%)<br>Ribosomal_S24e (4.6%)
5	1MBC	5YCI_A	4-43, 59-76, 101-118, 125-148	Globin (100%)
6	1NTI	AF-Q9D258-F1	1-14, 21-41, 62-86	COX1 (15.9%)<br>ACBP (11.1%)<br>H_PPase (8.7%)
7	1YYJ	6G7O_A	3-42, 56-79	Cytochrom_B562 (21.4%)<br>7tm_1 (16.6%)<br>Vinculin (5.9%)
30 human proteins (crystal structures and folding paths not determined by biological experiments)				
1	A0A1W2PQ64	AF-U7PWC0-F1	8-12, 18-37, 107-110, 134-139	adh_short_C2 (19.6%)<br>Pantoate_ligase(13.5%)<br>DLH (9.5%)
2	A0A5A2	AF-A0A5A2-F1	23-69, 83-113	V-set (50.7%)<br>C1-set (38.8%)<br>DUF1968 (10.4%)
3	A1L167	AF-A1L167-F1	1-9, 23-46, 52-116	UQ_con (77.1%)<br>TPR_8 (6.8%)<br>SHMT (5.1%)
4	A6NCE7	AF-Q9GZQ8-F1	13-37, 51-70, 79-83, 88-102, 109-115	ATG8 (31.1%)<br>PI3_PI4_kinase (17.4%)<br>Sel1 (5.4%)
5	A6NFH5	AF-A6NFH5-F1	7-15, 40-55, 80-131	Lipocalin (48.8%)<br>FGF (23.2%)<br>Lipoprotein_1 (8.4%)
6	A6NIZ1	AF-Q6TEN1-F1	2-25, 37-58, 75-104, 111-120, 128-151,154-168	Ras (100%)

7	A6NKH3	AF-P54051-F1	20-34, 66-91	RNA_pol_Rpb2_6 (38.5%)<br>TPR_8 (8.3%)<br>Sel1 (4.5%)
8	A6PVI3	AF-Q93594-F1	16-69, 76-120	RRM_1 (63.9%)<br>GTP_EFTU_D2 (4.1%)<br>Acyl_transf_1 (4.1%)
9	P01037	AF-P21460-F1	62-94, 106-124, 129-139	Cystatin (17.8%)<br>SnoaL_4 (5.9%)<br>Ring_hydroxyl_B (5.9%)
10	P09105	1ABW_A	5-36, 54-72, 93-137	Globin (95.7%)<br>Phycobilisome (3.8%)<br>Protoglobin (0.2%)
11	P0CE71	AF-Q9I8V0-F1	34-108	EF-hand_7 (59.0%)<br>EF-hand_5 (8.6%)<br>EF-hand_1 (4.5%)
12	P0CF74	AF-P01843-F1	7-14, 23-43, 65-73, 84-99	C1-set (50.9%)<br>V-set (49.1%)
13	P0CL84	AF-P0CL84-F1	19-35, 43-117	Arm (25.2%)<br>Arm_3 (17.5%)<br>HEAT_EZ (12.6%)
14	P0DI81	AF-Q9CQP2-F1	5-22, 60-110, 124-137	Peptidase_M4 (15.8%)<br>RVT_1 (10.3%)<br>Clat_adaptor_s (9.7%)
15	P36405	AF-Q9M9N1-F1	16-38, 51-99, 112-135, 153-181	Ras (66.0%)<br>Arf (28.2%)<br>MMR_HSR1 (2.9%)
16	P39877	AF-Q9WVF6-F1	20-32, 44-75, 97-125	Phospholip_A2_1 (59.9%)<br>COX1 (7.2%)<br>ABC_tran (6.3%)
17	P54849	AF-P54849-F1	2-24, 59-88, 95-120, 132-153	Acyl-CoA_dh_N (28.0%)<br>Vinculin (10.0%)<br>PMP22_Claudin (10.0%)
18	P60673	AF-P60673-F1	4-39, 85-112, 118-134	Profilin (14.9%)<br>GAF (10.4%)<br>Robl_LC7 (9.4%)
19	P61457	AF-P61459-F1	34-77, 87-103	Pyridoxal_deC (17.4%)<br>Aminotran_5 (13.0%)<br>Aminotran_1_2 (13.0%)
20	Q2M238	AF-Q2M238-F1	3-16, 22-38, 44-65, 75-99	CRM1_repeat (17.4%)<br>Arm (10.5%)<br>HEAT_EZ (8.7%)
21	Q4LDG9	AF-Q4LDG9-F1	19-41, 47-129, 139-150, 175-176	LRR_8 (68.6%)<br>LRR_adjacent (8.8%)<br>LRR_5 (3.8%)
22	Q5JXB2	AF-Q5JXB2-F1	34-87, 93-114, 134-148	UQ_con (99.3%)<br>zf-C3HC4_2 (0.7%)

23	Q5U5X0	AF-Q688I0-F1	2-42, 50-68	Ammonium_transp (26.9%)<br>Fe-ADH (10.6%)<br>ANAPC1 (6.7%)
24	Q6B0K9	1A3O_A	4-35, 92-112, 119-137	Globin (90.6%)<br>Phycobilisome (9.4%)
25	Q6IPR1	AF-Q6IPR1-F1	3-24,49-83	2014/3/3 (22.5%)<br>HEAT_EZ (9.8%)<br>TPR_8 (5.9%)
26	Q6ZSU1	AF-Q9DBX6-F1	2-15, 24-29, 38-69, 76-115	p450 (100%)
27	Q8N0U8	AF-Q8N0U8-F1	107-171	ABC_tran (17.8%)<br>FCH (13.8%)<br>COX1 (10.5%)
28	Q8N4G2	AF-Q8N4G2-F1	12-35, 47-68, 79-96, 114-132, 151-191	Ras (69.2%)<br>Arf (30.8%)
29	Q8NHR9	AF-Q5IRJ7-F1	3-41, 80-126	Profilin (17.0%)<br>GAF (12.5%)<br>Robl_LC7 (10.8%)
30	Q8TBF2	AF-Q8TBF2-F1	13-16, 28-106, 147-163, 173-183	1-cysPrx_C (40.2%)<br>AhpC-TSA (19.1%)<br>Redoxin (16.6%)

Table S6. The parameter descriptions in PAthreader.

Number of structure classes used in the second three-track alignment stage	N_{clu}	500
Number of templates to predict pDMScore	N_{pDM}	50
Number of templates used to identify intermediate	N_{t}	500
Threshold of ResFscore used to identify intermediate	I_{cut}	0.4
Weight of pDMScore in loss function	w	10
Infinitely small quantity in pDMScore	ε	0.001
Number of maxima of the distance profile	k_a	3
Number of maxima of the structure profile in the two three-track alignment stages	k_b	first stage: 3<br>second stage: 1
Threshold of inter-residue distance	λ	first stage: 10Å<br>second stage: 20Å

Table S7. Physical and geometric features extracted from templates.

Physical features	Amino acid properties	L×20	One-hot encoded amino acids.
		L×24	Blosum62 scores.
		L×7	Per amino-acid feature sets from Meiler.
	Secondary structures	L×4	Three state secondary structures given by DSSP solver.
	Rosetta energy	L×4	One-body terms: p_aa_pp, rama_prepro, omega, fa_dun.
		L×L×9	Two-body terms: fa_atr, fa_rep, fa_sol, lk_ball_wtd, fa_elec, hbond_bb_sc, hbond_sc, hbond_sr_bb, hbond_lr_bb.
Geometric features	Multi-distances	L×L×5	Inter-residue C_β to C_β distance map.<br>C_α to tip-atom distance map.<br>Tip-atom to C_α distance map.<br>Tip-atom to tip-atom distance map.<br>Sequence separation map.
	Orientations	L×L×6	cosine and sine of dihedral and planar angles defined by trRosetta.
	Backbone angles and lengths	L×6	Phi, Psi, and Omega angles.<br>Standardized length between backbone atoms.
	Voxelization	L×24×24 ×24×167	Voxelized each residue individually in the corresponding local coordinate frame defined by the backbone C, C_α, and N atoms.
	Ultrafast Shape Recognition	L×3	The first moment calculation of the distance sets at the three reference positions.

Supplementary References

1. Fu, L., Niu, B., Zhu, Z., Wu, S. & Li, W. CD-HIT: accelerated for clustering the next-generation sequencing data. *Bioinformatics* **28**, 3150-3152 (2012).
2. Steinegger, M. et al. HH-suite3 for fast remote homology detection and deep protein annotation. *BMC Bioinformatics* **20** (2019).
3. Zheng, W. et al. LOMETS3: integrating deep learning and profile alignment for advanced protein template recognition and function annotation. *Nucleic Acids Res* **50**, W454-W464 (2022).
4. Bai, Y., Sosnick, T.R., Mayne, L. & Englander, S.W. Protein folding intermediates: Native-state hydrogen exchange. *Science* **269**, 192-197 (1995).
5. Englander, S.W. & Mayne, L. The case for defined protein folding pathways. *Proc. Natl Acad. Sci. USA* **114**, 8253-8258 (2017).
