# Supplementary material for: Protein structure and folding pathway prediction based on remote homologs recognition using PAthreader
Source: Commun Biol. 2023 Mar 4;6:243. doi: 10.1038/s42003-023-04605-8 (PMC9985440; doi:10.1038/s42003-023-04605-8)
Supplement: Supplementary file 3 — Description of Additional Supplementary Files [file 42003_2023_4605_MOESM3_ESM.pdf]

Description of Additional Supplementary Files

File name: Supplementary Data 1

Description: The source data behind the Figures 2-3 in the paper

File name: Supplementary Data 2

Description: The source data behind the Figures 4 in the paper

File name: Supplementary Data 3

Description: The source data behind the Figures 5-6 in the paper
