# Supplementary material for: Protein structure and folding pathway prediction based on remote homologs recognition using PAthreader
Source: Commun Biol. 2023 Mar 4;6:243. doi: 10.1038/s42003-023-04605-8 (PMC9985440; doi:10.1038/s42003-023-04605-8)
Supplement: Supplementary file 7 — Reporting Summary [file 42003_2023_4605_MOESM7_ESM.pdf]

nature portfolio

Corresponding author(s): Guijun zhang

Last updated by author(s): Jan 31, 2023

Reporting Summary

Nature Portfolio wishes to improve the reproducibility of the work that we publish. This form provides structure for consistency and transparency in reporting. For further information on Nature Portfolio policies, see our Editorial Policies and the Editorial Policy Checklist.

Statistics

For all statistical analyses, confirm that the following items are present in the figure legend, table legend, main text, or Methods section.

n/a	Confirmed	
☐	☒	The exact sample size (*n*) for each experimental group/condition, given as a discrete number and unit of measurement
☐	☒	A statement on whether measurements were taken from distinct samples or whether the same sample was measured repeatedly
☐	☒	The statistical test(s) used AND whether they are one- or two-sided<br>*Only common tests should be described solely by name; describe more complex techniques in the Methods section.*
☒	☐	A description of all covariates tested
☐	☒	A description of any assumptions or corrections, such as tests of normality and adjustment for multiple comparisons
☐	☒	A full description of the statistical parameters including central tendency (e.g. means) or other basic estimates (e.g. regression coefficient) AND variation (e.g. standard deviation) or associated estimates of uncertainty (e.g. confidence intervals)
☐	☒	For null hypothesis testing, the test statistic (e.g. *F*, *t*, *r*) with confidence intervals, effect sizes, degrees of freedom and *P* value noted<br>*Give P values as exact values whenever suitable.*
☒	☐	For Bayesian analysis, information on the choice of priors and Markov chain Monte Carlo settings
☐	☒	For hierarchical and complex designs, identification of the appropriate level for tests and full reporting of outcomes
☐	☒	Estimates of effect sizes (e.g. Cohen's *d*, Pearson's *r*), indicating how they were calculated

Our web collection on statistics for biologists contains articles on many of the points above.

Software and code

Policy information about availability of computer code

Data collection	The online server of PAthreader are made freely available at http://zhanglab-bioinf.com/PAthreader/
Data analysis	The package of PAthreader are made freely available at GitHub (https://github.com/iobio-zjut/PAthreader)

For manuscripts utilizing custom algorithms or software that are central to the research but not yet described in published literature, software must be made available to editors and reviewers. We strongly encourage code deposition in a community repository (e.g. GitHub). See the Nature Portfolio guidelines for submitting code & software for further information.

Data

Policy information about availability of data

All manuscripts must include a data availability statement. This statement should provide the following information, where applicable:

- Accession codes, unique identifiers, or web links for publicly available datasets
- A description of any restrictions on data availability
- For clinical datasets or third party data, please ensure that the statement adheres to our policy

The authors declare that the data supporting the findings and conclusions of this study are available within the paper and its Supplementary Information file. The template library, structural profiles and trained distance profiles prediction model used by PAthreader are all available at http://zhanglab-bioinf.com/PAthreader/. Other data are available from the corresponding author upon reasonable requests.

Human research participants

Policy information about studies involving human research participants and Sex and Gender in Research.

Reporting on sex and gender	Sex and gender were not considered in study
Population characteristics	There were no human study participants in the study
Recruitment	n/a
Ethics oversight	n/a

Note that full information on the approval of the study protocol must also be provided in the manuscript.

Field-specific reporting

Please select the one below that is the best fit for your research. If you are not sure, read the appropriate sections before making your selection.

☒ Life sciences ☐ Behavioural & social sciences ☐ Ecological, evolutionary & environmental sciences

For a reference copy of the document with all sections, see nature.com/documents/nr-reporting-summary-flat.pdf

Life sciences study design

All studies must disclose on these points even when the disclosure is negative.

Sample size	We collected three data sets. (1) We constructed a comprehensive set of 551 non-redundant proteins with sequence identities < 30% from the SCOPe 2.07 database for template recognition. The resolutions of these proteins are less than 2 Å, and their lengths range from 120 to 700 residues. (2) We collected 186 proteins from 12 consecutive weeks (2022/04/01-2022/06/18) of CAMEO for protein structure modeling. (3) We collected 37 proteins for folding pathway prediction, 7 of which had been verified by biological experiments were collected from related reports, and the other 30 human proteins that had not been verified by biological experiments were randomly selected from UniProt release 2021_02.
Data exclusions	No data were excluded from the analyses.
Replication	All attempts at replication were successful.
Randomization	Based on the TM-score of the best template in PDB, we grouped the benchmark set into 4 subsets, i.e., 0-0.5, 0.5-0.7, 0.7-0.9 and 0.9-1.
Blinding	There is no blinding group in the template recognition experiment, as all proteins in our test have the deposited models in the PDB. The results of protein structure modeling were obtained through 12 consecutive weeks (2022/04/01-2022/06/18) of CAMEO blind testing, and it was validated by the CAMEO community after the blind testing. We predicted protein folding pathways for 37 proteins, in which the results of 7 proteins are almost consistent with those of biological experiments, and the other 30 human proteins have yet to be verified by biological experiments.

Reporting for specific materials, systems and methods

We require information from authors about some types of materials, experimental systems and methods used in many studies. Here, indicate whether each material, system or method listed is relevant to your study. If you are not sure if a list item applies to your research, read the appropriate section before selecting a response.

Materials & experimental systems

n/a	Involved in the study	
☒	☐	Antibodies
☒	☐	Eukaryotic cell lines
☒	☐	Palaeontology and archaeology
☒	☐	Animals and other organisms
☒	☐	Clinical data
☒	☐	Dual use research of concern

Methods

n/a	Involved in the study	
☒	☐	ChIP-seq
☒	☐	Flow cytometry
☒	☐	MRI-based neuroimaging
